# Intellectual disability and microcephaly associated with a novel *CHAMP1* mutation

**DOI:** 10.1038/s41439-021-00165-7

**Published:** 2021-08-17

**Authors:** Yuta Asakura, Hitoshi Osaka, Hiromi Aoi, Takeshi Mizuguchi, Naomichi Matsumoto, Takanori Yamagata

**Affiliations:** 1 Department of Pediatrics, Jichi Medical University, Tochigi, Japan; 2 Department of Human Genetics, Yokohama City University Graduate School of Medicine, Yokohama, Japan

**Keywords:** Neurodevelopmental disorders, Genetics of the nervous system

## Abstract

Mutations in a number of genes related to chromosomal segregation reportedly cause developmental disorders, e.g., chromosome alignment-maintaining phosphoprotein 1 (*CHAMP1*). We report on an 8-year-old Japanese girl who presented with a developmental disorder and microcephaly and carries a novel nonsense mutation in *CHAMP1*. Therefore, CHAMP1 mutation should be considered as a differential diagnosis of global developmental delay and microcephaly.

Chromosome alignment-maintaining phosphoprotein 1 (CHAMP1) is a zinc finger protein that functions in kinetochore-microtubule attachment and regulates chromosome segregation. Pathogenic variants of *CHAMP1* have been reported in patients with intellectual disability and other signs, such as microcephaly, muscular hypotonia, facial dysmorphism, and eye anomalies.^1–3^ Here, we report a novel nonsense mutation in *CHAMP1*, NM_001164144.2:c.1465C>T, p.(Gln489*).

The patient was an 8-year-old Japanese girl born to nonconsanguineous parents without neurological abnormalities, such as intellectual disability. She was delivered at full term by emergency Cesarean section because of umbilical cord entanglement without newborn asphyxia. Her birth weight was 2755 g (−0.47 standard deviation [SD]), her length was 46.8 cm (−1.5 SD), and her occipital frontal circumference was 31.8 cm (−0.9 SD). Her physical examination at birth was normal. She could control her head at 4 months of age and sit stably at 10 months. At 10 months of age, she developed acute encephalopathy after infection with respiratory syncytial virus and was treated with steroid pulse therapy in another hospital. She recovered without sequelae. However, since her recovery, developmental delay with acquired microcephaly (−2.4 SD) has become evident. Head magnetic resonance imaging was performed when she was 2 years old, and the scan showed mild atrophy of the cerebrum and cerebellum. She could walk independently at 3.5 years of age.

At 4 years of age, she came to our hospital because of global intellectual disability and acquired microcephaly. At the time of the visit, she had severe intellectual disability and could speak no meaningful words. A physical examination revealed no abnormalities. She was very friendly and displayed no dysmorphic facial features. A neurological examination showed normal muscle tone and tendon reflexes. No pyramidal tract signs, dystonia, or involuntary movements were observed. Her intelligence quotient according to a Tanaka-Binet test was 35. As the acute encephalopathy caused no brain magnetic resonance imaging abnormalities or sequelae, we searched for the cause of her intellectual disability and microcephalus. We initially suspected Angelman syndrome; however, a fluorescence in situ hybridization analysis failed to show a microdeletion at 15q11.2. After receiving informed consent, we performed whole-exome sequencing as previously described.^4^ We identified a novel *de novo* nonsense heterozygous variant, c.1465C>T, p.(Gln489*), in *CHAMP1* (Fig. 1).

**Fig. 1:**
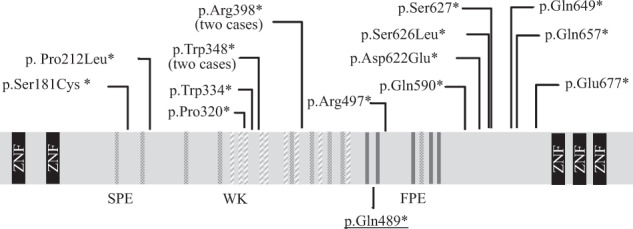
Diagram of CHAMP1 variants. Diagram of previously reported CHAMP1 variants (upper panel) and the mutation identified in this case (lower panel, underlined). CHAMP1 consists of five zinc-finger domains (ZNF) and several motifs; SPE (consensus: PxxSPExxK; dots), WK (SPxxWKxxP; diagonal lines), and FPE (FPExxK; grey bar).

In addition to *CHAMP1,*^1–3^ a number of mutations in genes related to chromosome alignment and/or spindle function, including Pogo transposable element-derived with ZNF domain (*POGZ*), tubulin gamma 1, dynein cytoplasmic 1 heavy chain 1, kinesin family member 5C (*KIF5C*), *KIF2A*, *KIF4A*, and centromere protein E, have been shown to cause various developmental disorders.^5–9^ CHAMP1 contains several repeat motifs, i.e., SPE, WK, and FPE motifs, and regulates kinetochore-microtubule attachment and chromosome alignment (Fig. 1). These motifs reportedly play an important role in spindle localization and kinetochore-microtubule interactions.^10^ The C-terminal region of *CHAMP1*, which contains the zinc-finger domain, has been predicted to be crucial for its proper localization to chromosomes and for mitotic spindle function.^1^ Functional studies using various deletion mutants of CHAMP1 have shown that lack of the C-terminal region of *CHAMP1* prevents proper chromosomal localization.^10^ Similar to the *CHAMP1* mutation in this case, all reported mutations are truncating mutations, i.e., nonsense or frameshift mutations that cause the loss of the C-terminal zinc-finger domain (Table 1). Although the biological mechanism that links *CHAMP1* mutations and intellectual disability is unknown, the pathogenic mechanism appears to be a result of the loss of the C-terminal region of *CHAMP1*. POGZ binds to the C-terminal region of CHAMP1 and is critical for proper chromosome segregation.^3,11^ Therefore, loss of the C-terminus may impair CHAMP1 chromosomal localization or/and its binding to other proteins, such as POGZ. Mitotic defects in neural progenitor cells may cause a decrease in the number of neural cells and defective neural development, resulting in intellectual disability.^3^

**Table 1:** *CHAMP1* variants and clinical presentation.

Patients	Sex	Mutation	Muscle hypotonia	Microcephaly	Delay in walking (>18m)	Impaired speech development	Eye anomalies	Brain MRI	Seizure	Abnormal behavior	Dysmorphic facial features	Reference
1	F	c.542_543delCT p.Ser181*	−	+	+	+	Strabismus	N/A	Febrile seizures	Skin-picking, rituals, food-foraging	N/A	Tanaka et al.^2^
2	F	c.635delC p. Pro212*	+	+	−	+	Hyperopia, astigmatism	Normal	−	Friendly, hand stereotypy, tacle hypersensitivity, sexual self-stimulation	+	Hempel et al.^1^
3	F	c.958_959delCC, p.Pro320*	N/A	−	+	+	N/A	non specific patchy signal abnormalities in right parieto-occipital subcortical white matter, bulky corpus callosum autistic behavior	−	+	+	Isdor et al.^3^
4	M	c.1002G>A p.Trp334*	N/A	−	+	+	Hyperopia, strabismus	Normal	−	-	−	Isdor et al.^3^
5	F	c.1043G>A, p.Trp348*	N/A	+	+	+	Hyperopia, astigmatism	thickening of the corpus callosum, subtle hypoplasia of the left temporal lobe	−	+	+	Isdor et al.^3^
6	F	c.1044delG p.Trp348*	+	+	−	+	−	Hypoplastic corpus callosum	−	Aggressive, occasionally self injurious	N/A	Tanaka et al.^2^
7	M	c.1192C>T p.Arg398*	+	+	+	+	Strabismus, hyperopia	Normal	−	Friendly	+	Hempel et al.^1^
8	F	c.1192C>T p.Arg398*	+	−	−	+	Hyperopia, astigmatism	Normal	−	Friendly	+	Hempel et al.^1^
9	F	c.1465C>T, p.Gln489*	−	+	+	+	−	mild atrophy of the cerebrum and cerebellum	−	Friendly	−	This case
10	F	c.1489C>T, p.Arg497*	N/A	−	+	+	Amblyopia	N/A	N/A	−	+	Isdor et al.^3^
11	M	c.1768C>T p.Gln590*	+	+	+	+	Impaired	delayed myelination	Fromtotemporal epilepsy	Hand stereotypy, friendly	+	Hempel et al.^1^
12	M	c.1866_1867delCA p.Asp622*	+	+	+	+	Strabismus, hyperopia	Mild brain atrophy and cerebellar cortical dysplasia	−	Hand stereotypy, friendly	+	Hempel et al.^1^
13	F	c.1876_1877delAG; p.Ser626*	N/A	+	+	+	Hyperopia, astigmatism	arachnoid cyst	+	+	+	Isdor et al.^3^
14	M	c.1880C>G p.Ser627*	N/A	+	+	+	Hyperopia, astigmatism, strabismus	Normal	−	−	+	Isdor et al.^3^
15	F	c.1945C>T p.Gln649*	+	−	+	+	Ocuular albinism	Normal	−	ADD/ADHD	N/A	Tanaka et al.^2^
16	F	c.1969C>T p.Gln657*	+	+	+	+	Strabismus	Mild decreased white matter	Seizure at 3 yo	Inapropriate laughter	N/A	Tanaka et al.^2^
17	F	c.2029G>T p.Glu677*	+	+	+	+	Strabismus	Mild cerebellar atrophy	−	Hyperactivity	N/A	Tanaka et al.^2^

+ present, − absent, *N/A* not available, *ADD/ADHD* attention deficit disorder/attention deficit hyperactivity disorder.

The clinical presentation of *CHAMP1* mutation includes intellectual disability (17/17), motor development delay (14/17), facial dysmorphism (10/12), eye anomalies (14/16), and microcephaly (12/17). For some individuals, anomalies are evident on magnetic resonance imaging scans of the brain (9/15) and abnormal behavior varies from mild to severe (14/17) (Table 1).^1–3^ Some patients present with a feeding disorder. Considering these symptoms, Angelman syndrome and Prader-Willi syndrome are the most important differential diagnoses.^1^

In conclusion, we report a novel nonsense mutation in *CHAMP1*, c.1465C>T, p.(Gln489*). The patient had intellectual disability, motor development delay, and microcephaly but no facial dysmorphism or eye anomalies. *CHAMP1* mutation should be considered when a patient presents with global developmental delay and microcephaly.

## HGV database

The relevant data from this Data Report are hosted at the Human Genome Variation Database at 10.6084/m9.figshare.hgv.3081.
